# Functional differentiation and spatial-temporal co-expression networks of the NBS-encoding gene family in Jilin ginseng, *Panax ginseng* C.A. Meyer

**DOI:** 10.1371/journal.pone.0181596

**Published:** 2017-07-20

**Authors:** Rui Yin, Mingzhu Zhao, Kangyu Wang, Yanping Lin, Yanfang Wang, Chunyu Sun, Yi Wang, Meiping Zhang

**Affiliations:** 1 College of Life Science, Jilin Agricultural University, Changchun, Jilin, China; 2 The Center of Ginseng Germplasm Research, Development and Utilization, Changchun, Jilin, China; 3 College of Chinese Medicinal Materials, Jilin Agricultural University, Changchun, Jilin, China; New Mexico State University, UNITED STATES

## Abstract

Ginseng, *Panax ginseng* C.A. Meyer, is one of the most important medicinal plants for human health and medicine. It has been documented that over 80% of genes conferring resistance to bacteria, viruses, fungi and nematodes are contributed by the nucleotide binding site (NBS)-encoding gene family. Therefore, identification and characterization of NBS genes expressed in ginseng are paramount to its genetic improvement and breeding. However, little is known about the NBS-encoding genes in ginseng. Here we report genome-wide identification and systems analysis of the NBS genes actively expressed in ginseng (*PgNBS* genes). Four hundred twelve *PgNBS* gene transcripts, derived from 284 gene models, were identified from the transcriptomes of 14 ginseng tissues. These genes were classified into eight types, including TNL, TN, CNL, CN, NL, N, RPW8-NL and RPW8-N. Seven conserved motifs were identified in both the Toll/interleukine-1 receptor (TIR) and coiled-coil (CC) typed genes whereas six were identified in the RPW8 typed genes. Phylogenetic analysis showed that the *PgNBS* gene family is an ancient family, with a vast majority of its genes originated before ginseng originated. In spite of their belonging to a family, the *PgNBS* genes have functionally dramatically differentiated and been categorized into numerous functional categories. The expressions of the across tissues, different aged roots and the roots of different genotypes. However, they are coordinating in expression, forming a single co-expression network. These results provide a deeper understanding of the origin, evolution and functional differentiation and expression dynamics of the NBS-encoding gene family in plants in general and in ginseng particularly, and a NBS gene toolkit useful for isolation and characterization of disease resistance genes and for enhanced disease resistance breeding in ginseng and related species.

## Introduction

Ginseng, *Panax ginseng* A.C. Meyer (2*n* = 4*x* = 48), is a traditional Chinese medicinal herb, a perennial of the *Araliaceae* family and has been cultivated in China for over 2,000 years. Studies have shown that ginseng possesses several important biological functions for human health, such as recovery and promotion of vitality, improvement of immune and metabolism systems, regulation of central nervous system, etc. [1, 2]. Ginseng native to the Province of Jilin, China, is often known as Jilin ginseng and estimated to produce 85% and 70% of the ginseng of China and the world, respectively. However, it is being subjected to numerous diseases that threaten the continued production of Jilin ginseng. Therefore, it is imperative to improve its disease resistance to continue and secure ginseng production. Nevertheless, because it is perennial, ginseng breeding using traditional methods is a great challenge. Molecular breeding promises to significantly enhance ginseng genetic improvement and breeding.

Plants have evolved different strategies to protect themselves from pathogens. One of the most important and well-studied mechanisms in which plants defense pathogens is based on disease resistance (R) genes whose products recognize pathogen avirulence (*Avr*) gene directly or indirectly [3]. This gene-for-gene mechanism may activate signal transduction cascades that turn on complex defense responses against pathogens [4]. Studies have documented that most of the cloned R genes that were shown to confer resistance to pathogens encode proteins containing a nucleotide binding site (NBS) domain and a leucine-rich repeat (LRR) domain [5]. These genes have often been referred as to the NBS genes. The NBS domain of the R genes is a region of approximately 300 amino acids extending from the P-loop to the MHDV motif [6]. This domain is a signaling domain responsible for the binding and hydrolysis of ATP and GTP. The LRR domain is devoted to protein-protein interactions. Under a diversifying selection, the LRR domain evolves different binding specificities, which may play a vital role in defining pathogen recognition specificity [4].

The NBS genes have been classified into two major groups, based on their protein structures at N-termini [7]. One group has a Toll/interleukin-1 receptor (TIR) domain in the N-terminal region and the gene members of this group are usually defined TIR-NBS-LRR or TNL genes. The other group has a coiled-coil (CC) structure in the N-terminal region and the gene members of this group are often defined CC-NBS-LRR or CNL genes. Both groups contain five conserved and strictly ordered motifs in the NBS domain, including P-loop, kinase-2, kinase-3a, GLPL, and MHDL [8–11]. Phylogenetic analyses positioned the CNL and TNL genes in separated clades of the NBS gene family phylogenic tree [7, 9, 12].

NBS genes have been genome-wide identified and characterized in several plant species, including *Arabidopsis thaliana* [12, 13], rice [12, 14], cucumber [15], poplar [16], papaya [16], potato [11], *Lotus japonica* [17], *Brassica rapa* [17], soybean [17] and pepper [18]. These studies showed that NBS-LRR genes appear as a large superfamily consisting of hundreds of gene members, constituting 0.20% - 1.76% of the genes of the species [16]. However, little is known about the NBS genes in ginseng and their evolution, expressions and potential functions.

We previously sequenced and characterized the transcriptomes and expression profiles of the genes expressed in fourteen tissues of four-year-old plants, four different-year-old roots and four-year-old roots of 42 genotypes of Jilin ginseng [19]. From the transcriptomes of the 14 tissues, we assembled 248,992 transcript unigenes because studies have documented that different transcripts alternatively spliced from a gene are translated into different proteins potentially having different functions [20]. These expressed gene sequences and their expression profiles have provided resources necessary for genome-wide analysis of the NBS genes actively expressed in ginseng. In this study, we identified and categorized the NBS genes, analyzed their origin and evolution, constructed their phylogeny and characterized their expressions and networks in 14 tissues, four differently aged roots and four-year-old roots of 42 genotypes. These results have provided a deep insight into the NBS genes and the mechanisms underlying their function and evolution in ginseng and thus will facilitate disease resistance genetic improvement and breeding in ginseng and related species.

## Materials and methods

### Identification of the *PgNBS* genes expressed in ginseng

The 248,992 transcript unigenes previously generated from the transcriptomes of 14 tissues of four-year-old Jilin Ginseng cv. Damaya plants (S1 Fig) [19] were used for this study. Identification of NBS genes expressed in ginseng was as described for identification of the NBS genes in soybean and *L*. *japonicas* [17, 21].

First, search was performed for possible homologs to NBS genes in the Jilin ginseng transcript unigene database by TBLASTN with the amino acid sequence of the NB-ARC domain (Pfam: PF00931) as a query. A threshold of 1.0E-04 was determined empirically and used for the search to filter out most of the spurious hits. Second, the candidate NBS genes were further subjected to the BLASTn search at an e-value of 1.0E-05 using their nucleotide sequences as queries. Third, the Blast2GO program [22] was used to filter out the potential spurious hits using an e-value of ≤ 1.0E-05. Finally, the transcripts identified as above that had same gene models were considered to be derived from a single NBS gene. The NBS genes identified herein were named *PgNBS001* –*PgNBS284*, with a digital suffix (e.g., -01) for a transcript of a *PgNBS* gene (S1 Table).

Furthermore, the *PgNBS* genes were analyzed using the Pfam database (http://pfam.janelia.org/) to verify whether they encode TIR, RPW8, NBS or LRR motifs. Because the Pfam program did not predict the CC structure of NBS genes, the COILSn program (http://www.ch.embnet.org/software/COILS_form.html) was used to detect the CC structures of the *PgNBS* genes at a threshold of 0.9 [23].

### *In silico* annotation and functional categorization

Because different transcripts of a gene may have different biological functions [20], the *PgNBS* gene transcripts were subjected to annotation (S2 Table). The putatively encoding proteins of the *PgNBS* transcripts were searched against the Arabidopsis protein database in the Arabidopsis Information Resource (TAIR, http://www.arabidopsis.org), the Swiss-Prot protein database (http://www.expasy.ch/sprot) and the NCBI non-redundant protein (Nr) database (http://www.ncbi.nlm.nih.gov) using the BLASTX algorithm at an e-value of 1.0E-10. The *PgNBS* genes were then functionally categorized using gene ontology (GO) term by Blast2GO [22].

### Phylogenetic analysis

To determine the origin and evolution of the *PgNBS* genes, the *PgNBS* genes having complete NBS domains were extracted and aligned using the ClustalW program [24]. The *PgNBS* genes with poor sequence alignments were manually removed. Consequently, 67 *PgNBS* genes, including 9 CN-, 2 CNL-, 34 N-, 7 NL-, 4 RPW8-N-, 9 TN- and 2 TNL-type NBS genes (S3 Table), were selected to construct the phylogenetic tree of the *PgNBS* gene family. To estimate the origin of the *PgNBS* genes, a selection of the NBS-encoding R genes previously cloned and known to confer disease resistance from a group of species of known position in a seed plant phylogenetic tree were included as references (S4 Table) [25]. The conserved sequences from the P-loop to GLPL of the *PgNBS* proteins were aligned and used to construct the phylogenetic tree of the *PgNBS* gene family using the neighbor-joining method by MEGA 5 [26]. The confidence of the tree was estimated using 1,000 bootstrap replications, the model of Poisson correction was applied and the missing data were treated by Pairwise Deletion of the gaps.

### Protein conserved motif structure analysis

To further characterize the structure diversity of the predicted proteins encoded by the *PgNBS* genes, the TIR-, CC- and RPW8-NBS genes were selected and the amino acid sequences of their NBS domains and the N termini were subjected to motif analysis. The conserved motifs in these representative genes were then analyzed using the MEME online program (http://meme.sdsc.edu/meme/website/intro.html) [24].

### Expression profile analysis

The expression profiles of the *PgNBS* transcripts in 14 tissues of four-year-old plants (S5 Table), four different year-old roots (S6 Table) and four-year-old roots of 42 Jilin ginseng farmers’ cultivars (S7 Table) were extracted from the expressed profile database of their transcriptomes previously developed [19]. The *PgNBS* transcripts were then subjected to heatmap construction and co-expression network analysis. The expression heatmaps were constructed using the R programming language and software (http://www.r-project.org/) and the co-expression networks were constructed and visualized using the BioLayout Express^3D^ software [27].

## Results

### Identification of *PgNBS* genes

Four hundred and twelve *PgNBS* gene transcripts were identified (S1 Table) from the 248,992 transcript unigenes of Jilin ginseng [19]. These *PgNBS* transcripts were derived from 284 gene models. These 284 *PgNBS* genes were classified into eight types, based on their N terminal, C terminal and LRR domains (Table 1). Of the 284 *PgNBS* genes, 11 (3.87%) were identified to have the TIR domains at the N-termini, two of which contain the LRR domain (coded by TNL) and nine do not (coded by TN). Sixteen of the *PgNBS* genes (5.63%) have the CC motifs at the N-termini, two of which have the LRR domain (coded by CNL) and 14 do not (coded by CN). Five *PgNBS* genes (1.76%) have the RPW8 domain, which is known as *Arabidopsis* resistance to powdery mildew 8 (RPW8) [28]. Of these five RPW8 *PgNBS* genes, one has the LRR domain (coded by RPW8-NL) and four do not (coded by RPW8-N). Thirteen of the *PgNBS* genes (4.58%) possess the NBS-LRR domain (coded by NL) and 239 (84.15%) only have the NBS domain (coded by N). S2 Table shows the details of the genes classification.

**Table 1:** Classification of the *PgNBS* genes based protein structures.

Predicted protein domains	Letter code	No. of genes	Percentage	No. of transcripts
TIR-NBS-LRR	TNL	2	0.70	3
CC-NBS-LRR	CNL	2	0.70	3
TIR-NBS	TN	9	3.17	19
CC-NBS	CN	14	4.93	35
NBS	N	239	84.15	316
NBS-LRR	NL	13	4.58	26
RPW8-NBS-LRR	RPW8-NL	1	0.35	1
RPW8-NBS	RPW8-N	4	1.41	9
Total		284	100	412

### Annotation, functional categorization and pathway mapping of *PgNBS* genes

**Fig 1:**
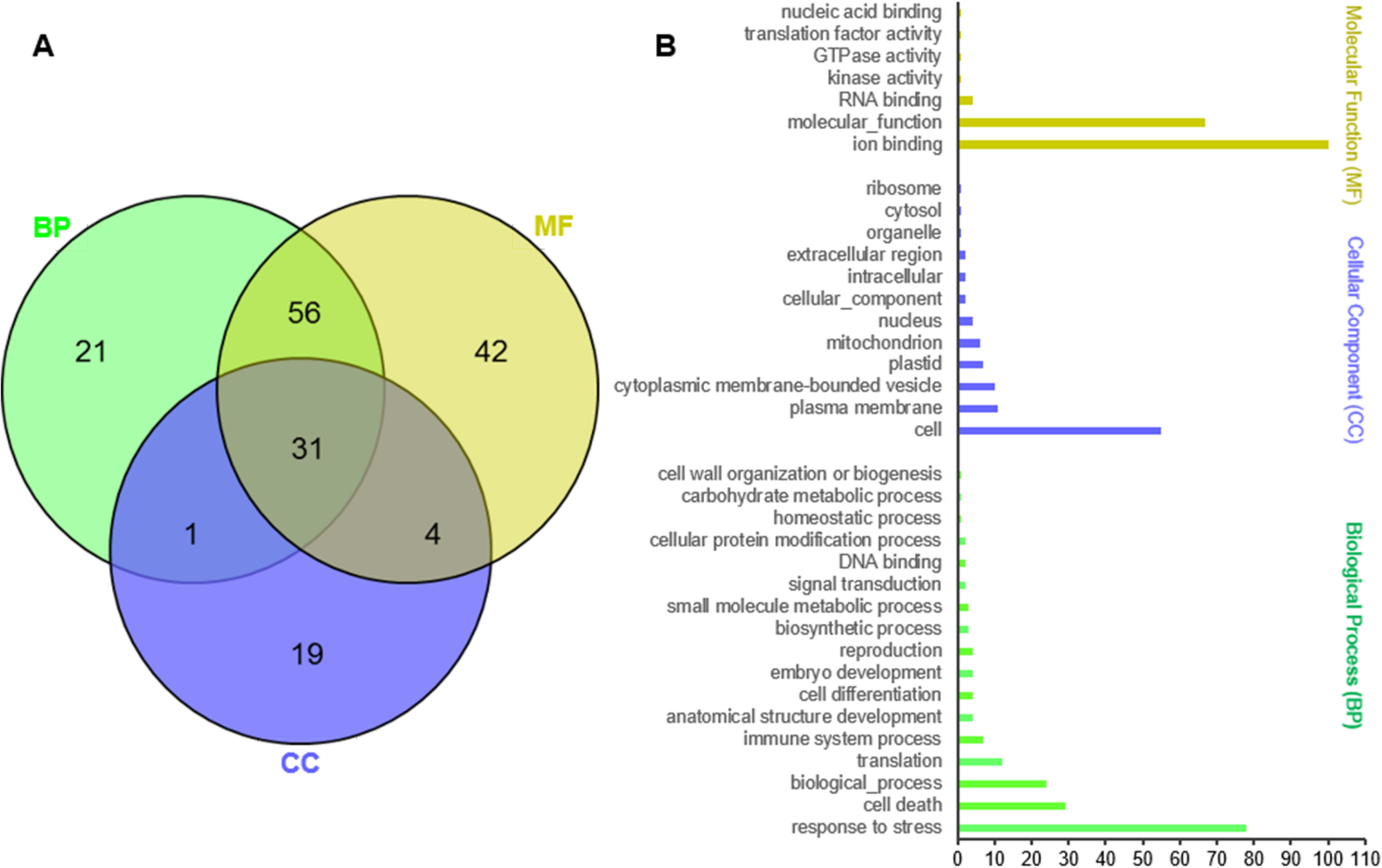
Functional categorization of the *PgNBS* gene transcripts by gene ontology (GO). (A) Venn diagram of the *PgNBS* gene transcripts among the biological process (BP), molecular function (MF) and cellular component (CC) functional categories. (B) The *PgNBS* gene transcripts were categorized into 36 functional categories (Level 2), including seven MF functional categories, 12 CC functional categories and 17 BP functional categories.

To infer the potential biological functions of the *PgNBS* genes, we first annotated the gene transcripts, functionally categorized them *in silico* and mapped to pathways. Of the 412 *PgNBS* transcripts, 172 were annotated, based on sequence similarities to proteins in TAIR database and assigned to GO terms (S2 Table). The remaining 238 *PgNBS* gene transcripts could not be annotated. One hundred thirty-three, 109 and 55 of the 174 *PgNBS* gene transcripts were annotated to molecular function (MF), biological process (BP) and cellular compound (CC), respectively (Fig 1A). Of these gene transcripts, only 31 (17.8%) had functions in all three categories. Moreover, the genes of each category were further categorized into multiple functional subcategories (Level 2), even though a majority of the *PgNBS* genes categorized into Molecular_Function, Ion Binding, Cell and Response to Stress (Fig 1B). These results indicate that the *PgNBS* gene family has been dramatically differentiated in function, since its gene members originated. Nevertheless, none of them was mapped to the KEGG metabolic pathways.

### NBS domain analysis of the *PgNBS* putative proteins

**Fig 2:**
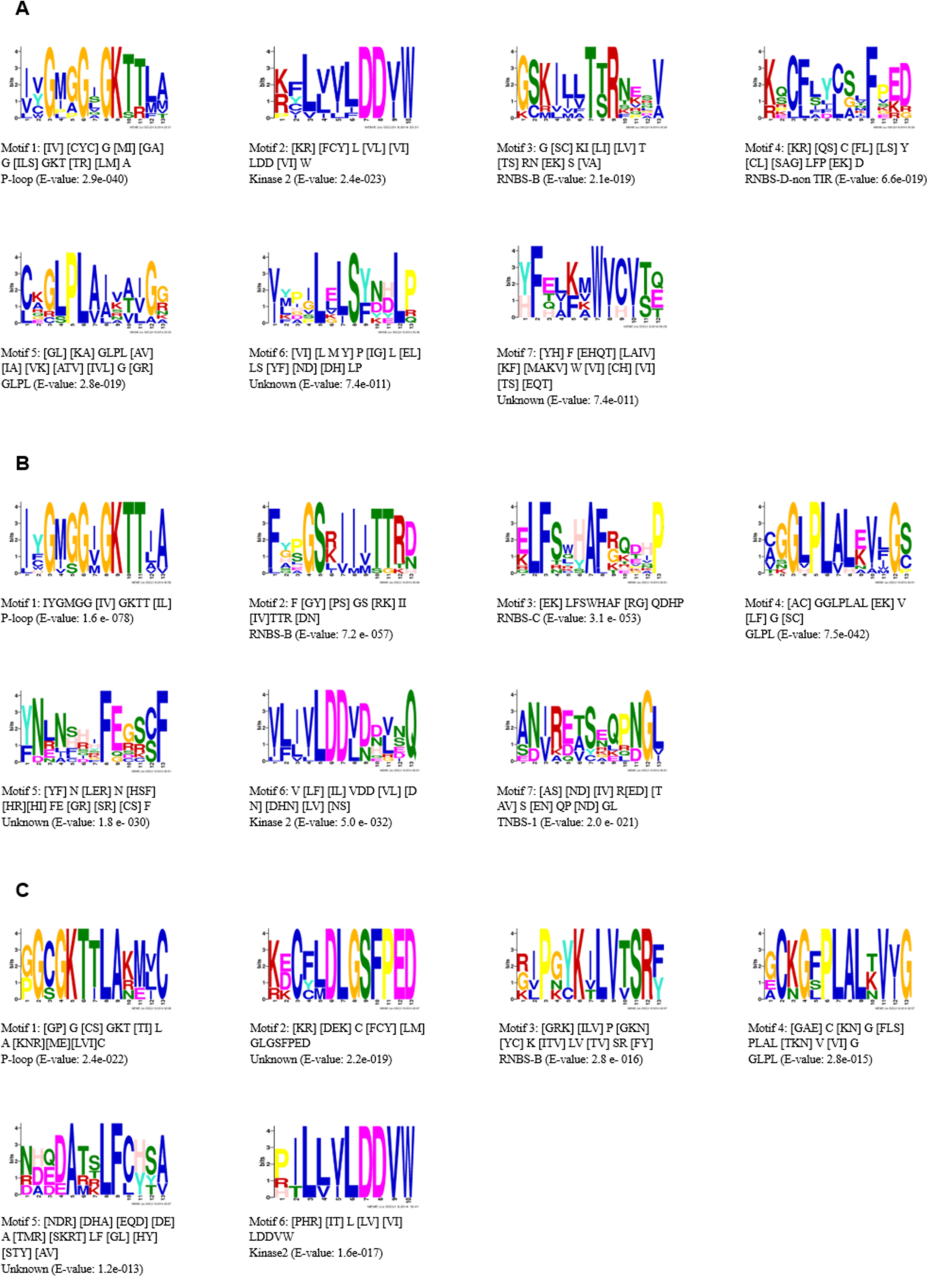
Conserved amino acid motifs in the NBS domain of the *PgNBS* gene proteins identified using the MEME 4.0 software. (A) Sequence logo of seven prominent conserved motifs in the NBS domain of the CC type. (B) Sequence logo of seven prominent conserved motifs in the NBS domain of the TIR type. (C) Sequence logo of six prominent conserved motifs in the NBS domain of the RPW8 type.

Because eight major motifs, P-loop, RNBS-A, Kinase-2, RNBS-B, RNBS-C, GLPL, RNBS-D, and MHDV, have been identified in the NBS region of the known NBS-encoding genes in plants [11], we analyzed the *PgNBS* genes using these motifs. MEME analysis revealed that seven of the eight conserved motifs (except for the MHDV motif) exist in the TIR- and CC-typed *PgNBS* genes (Fig 2A and 2B), while only six exist in the RPW8-typed *PgNBS* genes (Fig 2C). Four of these conserved motifs, including P-loop, TNBS-B, Kinase-2 and GLPL, exist in all TIR-, CC- and RPW8-typed *PgNBS* genes (Fig 2).

In the CC-typed *PgNBS* genes, the RNBS-D motif was specially identified in their NBS domain and two of their other conserved motifs (Fig 2A, Motifs 6 and 7) are dramatically different from the motifs that previously reported. In addition, the proteins of two *PgNBS* genes (*PgNBS027* and *PgNBS051-01*) were shown to be weakly conserved in both P-loop and GLPL motifs, and the proteins of some *PgNBS* genes lack one or two motifs in the NBS region. For instance, the protein of *PgNBS004* lacks the Kinase-2 motif and that of *PgNBS034* lacks the GLPL motif.

In the TIR-typed *PgNBS* genes, the RNBS-C and TNBS-1 motifs were specially identified in their NBS domain. Moreover, some amino acids were diverged from those of the CC-typed *PgNBS* genes in some motifs. For example, in the TNBS-B domain a conserved amino acid sequence is TTR for TIR-typed *PgNBS* genes, while it is TTR or TSR for CC-typed *PgNBS* genes. In the Kinase-2 motif, a conserved amino acid sequence was DDVD or DDVN for TIR-typed *PgNBS* genes, while it is DDVW for CC-typed *PgNBS* genes (Fig 2B). The proteins of some TIR-typed *PgNBS* genes were also weakly conserved in the NBS domain. For example, those of *PgNBS038-01* and *PgNBS040* were weakly conserved in the Kinase-2 motif.

In the RPW8-typed *PgNBS* genes, except for the four conserved motifs described above, two highly conserved motifs (motif5:[KR][DEK]C[FCY][LM]DLGSFPED and motif6:[NDR][DHA][EQD][DE]A[TMR][SKRT]LF[CL][HY][STY][AV]) were specially identified (Fig 2C).

### N-terminal region analysis of *PgNBS* putative proteins

**Fig 3:**
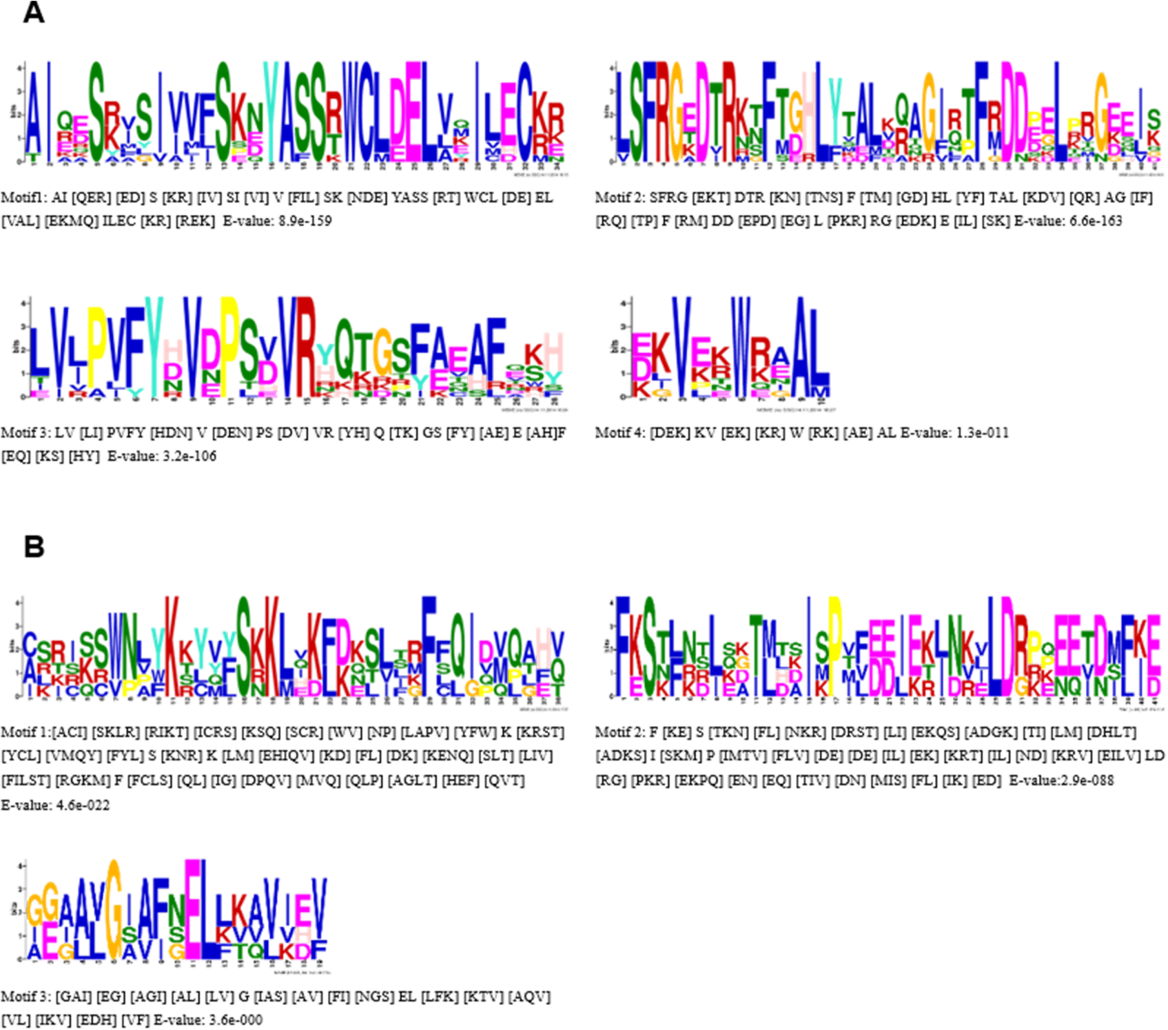
Conserved amino acid motifs in the N terminal region of the *PgNBS* gene proteins identified using the MEME 4.0 software. (A) Sequence logo of prominent conserved motifs in the N terminal region of the TIR type. (B) Sequence logo of prominent conserved motifs in the N terminal region of the RPW8 type.

The N-terminal region often ranges from 150 to 250 amino acids from the start of the coding region to the beginning of the P-loop of the NBS domain. We analyzed the N-terminal regions of the putative proteins in different typed *PgNBS* genes separately. No consistent motifs were identified in the CC-typed *PgNBS* genes, whereas four and three distinct conserved motifs were identified in the N-terminal regions of TIR- and RPW8-typed *PgNBS* genes, respectively (Fig 3).

### Origin, evolution and phylogeny of *PgNBS* genes

Studies showed that the NBS domains of the NBS genes are highly conserved and have been used to construct phylogenetic trees for the NBS gene family in several species [15, 29]. Therefore, we investigated the origin, evolution and phylogeny of the *PgNBS* gene family using the amino acid sequences of this domain. Analysis showed that 67 of the *PgNBS* genes have a complete NBS domain and therefore, was used for this experiment. These 67 *PgNBS* genes included 9 CN-, 2 CNL-, 34 N-, 7 NL-, 4 RPW8-N-, 9 TN- and 2 TNL-typed *PgNBS* genes (S3 Table). Moreover, 18 previously cloned R genes were selected from a selection of species that were able to position *P*. *ginseng* in the phylogenic tree of seed plants [25] and used as references to estimate the origin and evolution of the *PgNBS* gene family. These 18 R genes were from *Arabidopsis thaliana*, *Helianthus annuus*, *Cicer arietinum*, *Camelina sativa*, *Oryza sativa* and *Solanum tuberosum*, including 10 TN-, 5 CN- and 3 RPW8-N-typed R genes (S4 Table). The amino acid sequences of the NBS domains were predicted, aligned using the region between the P-loop and GLPL motifs and used to construct the phylogenetic tree of the *PgNBS* gene family by the Neighbor-Joining method.

**Fig 4:**
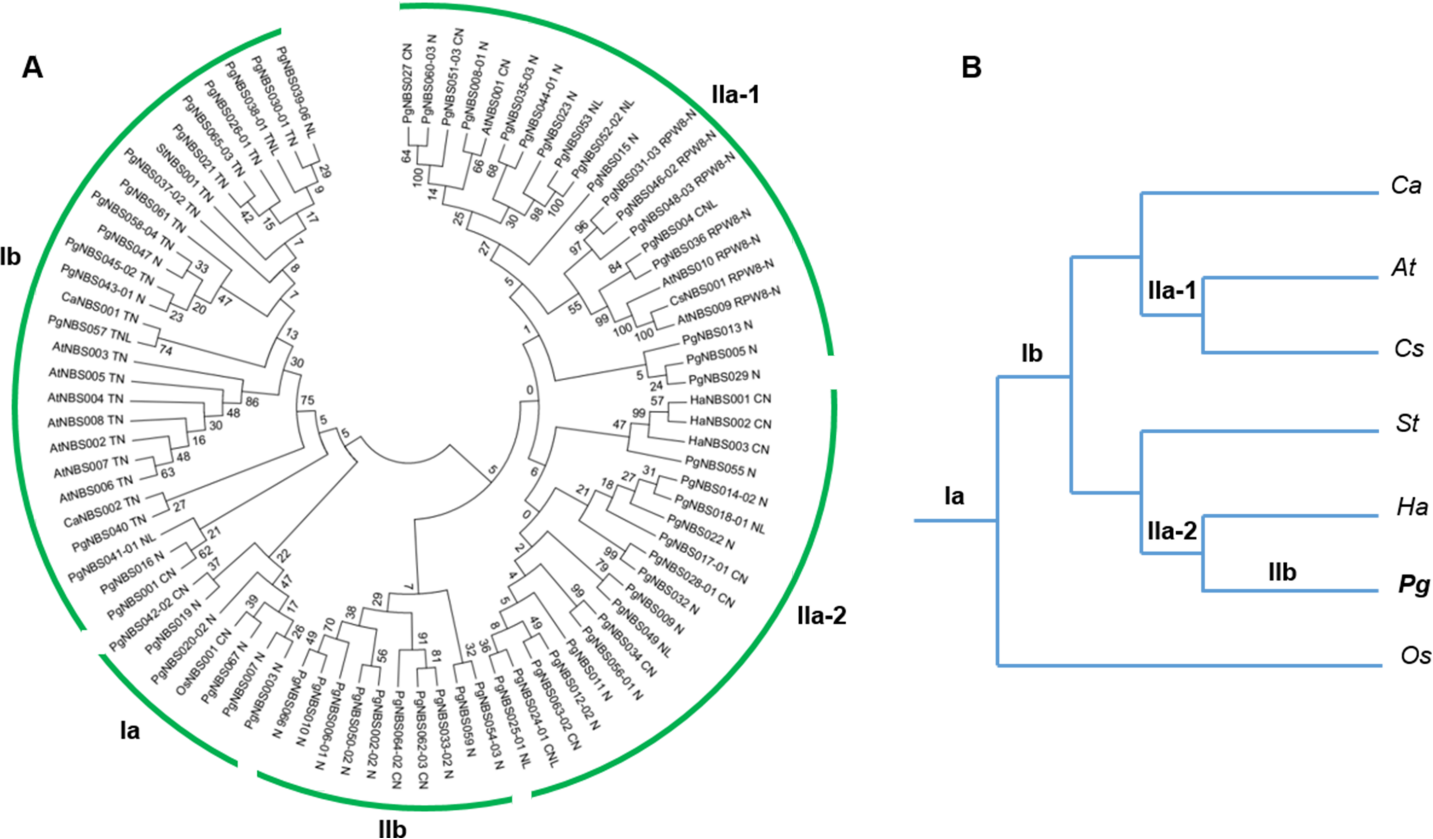
Origin, evolution and phylogeny of the *PgNBS* gene family. (A) Phylogeny of the *PgNBS* gene family. The number nearby each clade or branch indicates the bootstrap confidence derived from 1000 replications. (B) Phylogeny of *P*. *ginseng* and selected plant species as references for inference of origin and evolution of the *PgNBS* gene family. The phylogenic tree of the species was from [25]. *Pg*, *Panax ginseng*; *At*, *Arabidopsis thaliana*; *Ca*, *Cicer arietinum*; *Cs*, *Camelina sativa*; *Ha*, *Helianthus annuus*; *Os*, *Oryza sativa; St*, *Solanum tuberosum*.

Fig 4 shows the origin, evolution and phylogeny of the *PgNBS* gene family resulted from this analysis. The *PgNBS* gene family was classified into two large clades, I and II. Clade I was further classified into two subclades, Ia and Ib. Clade II was classified into two subclades, IIa and IIb, and Subclade IIa was then classified into two clusters, IIa-1 and IIa-2 (Fig 4A). According to the phylogenetic tree of *P*. *ginseng* and the reference species (Fig 4B), Subclade Ia was originated before monocot plants (*O*. *sativa*) originated because it includes the NBS genes that were not only from *P*. *ginseng*, but also from *O*. *sativa*. Subclade Ib originated before the dicot plants split. Subclade IIb was originated most recently, after *P*. *ginseng* split from *H*. *annuus*. Therefore, the *PgNBS* gene family is an ancient, but continuously evolving gene family.

### Expression characteristics of *PgNBS* gene transcripts in different tissues

**Fig 5:**
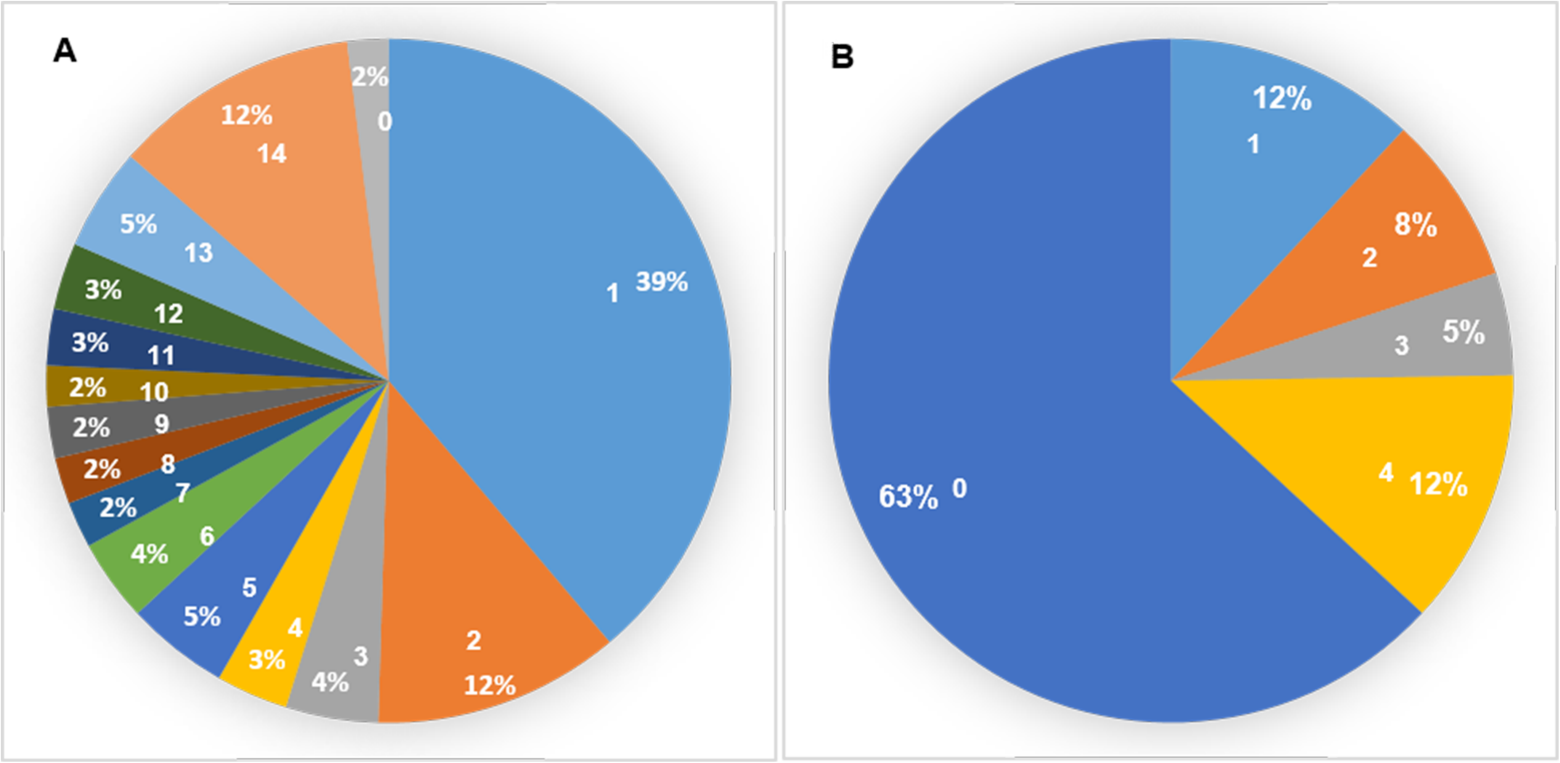
Expression of the *PgNBS* gene transcripts in ginseng. (A) Expression of the *PgNBS* gene transcripts in different numbers of tissues. (B) Expression of the *PgNBS* gene transcripts in differently-aged roots. The percentages of each part of the pie indicates the percentage of the 412 *PgNBS* gene transcripts; the number behind the percentage in each part of the pie indicates the number of the 14 tissues (A) or the number of four aged roots (B) in which the gene transcripts expressed.

To determine the activity characteristics of the *PgNBS* genes in ginseng, we first profiled the expressions, determined the GO functional categories and constructed the expression heatmap and co-expression network of the gene transcripts in 14 tissues of four-year-old ginseng plants (S1 Fig). The gene transcripts were analyzed because different transcripts likely have different biological functions [20]. We found that of the 412 *PgNBS* transcripts, 403 expressed in at least one of these tissues and their expression profiles varied dramatically among tissues (Fig 5A).

Only 49 of the *PgNBS* gene transcripts expressed in all 14 tissues analyzed, accounting for 12%, while 160 of them specifically expressed in only one tissue, accounting for 39%, suggesting the tissue specificity of their expressions. The remaining 194 *PgNBS* gene transcripts (47%) expressed in two to thirteen of the 14 tissues analyzed. Among the 14 tissues, the number of gene transcripts expressed in each tissue varied from 84 to 336, with an average of 147. The largest number (336) of the 412 *PgNBS* gene transcripts expressed in fruit pedicel (L), while the least number of the transcripts (84) expressed in seed (N). From 118 to 160 of the transcripts expressed in the remaining 12 tissues, including fiber roots (A), leaf blade (J), arm root (F), leg root (B), leaflet pedicel (I), main root epiderm (C), fruit peduncle (K), main root cortex (D), rhizome (E), stem (G), leaf peduncle (H) and fruit flesh (M) in ascending order (S5 Table). These results indicated that the numbers of *PgNBS* gene transcripts expressed in each tissue varied greatly, with a coefficient of variation (CV) = 39.2%. However, if the 336 gene transcripts expressed in fruit pedicel that dramatically differed from those expressed in the 13 other tissues were excluded from the analysis, the variation of the expressed transcripts in each tissue ranged from 84 to 160, with an average of 132 and CV = 15.2%.

**Fig 6:**
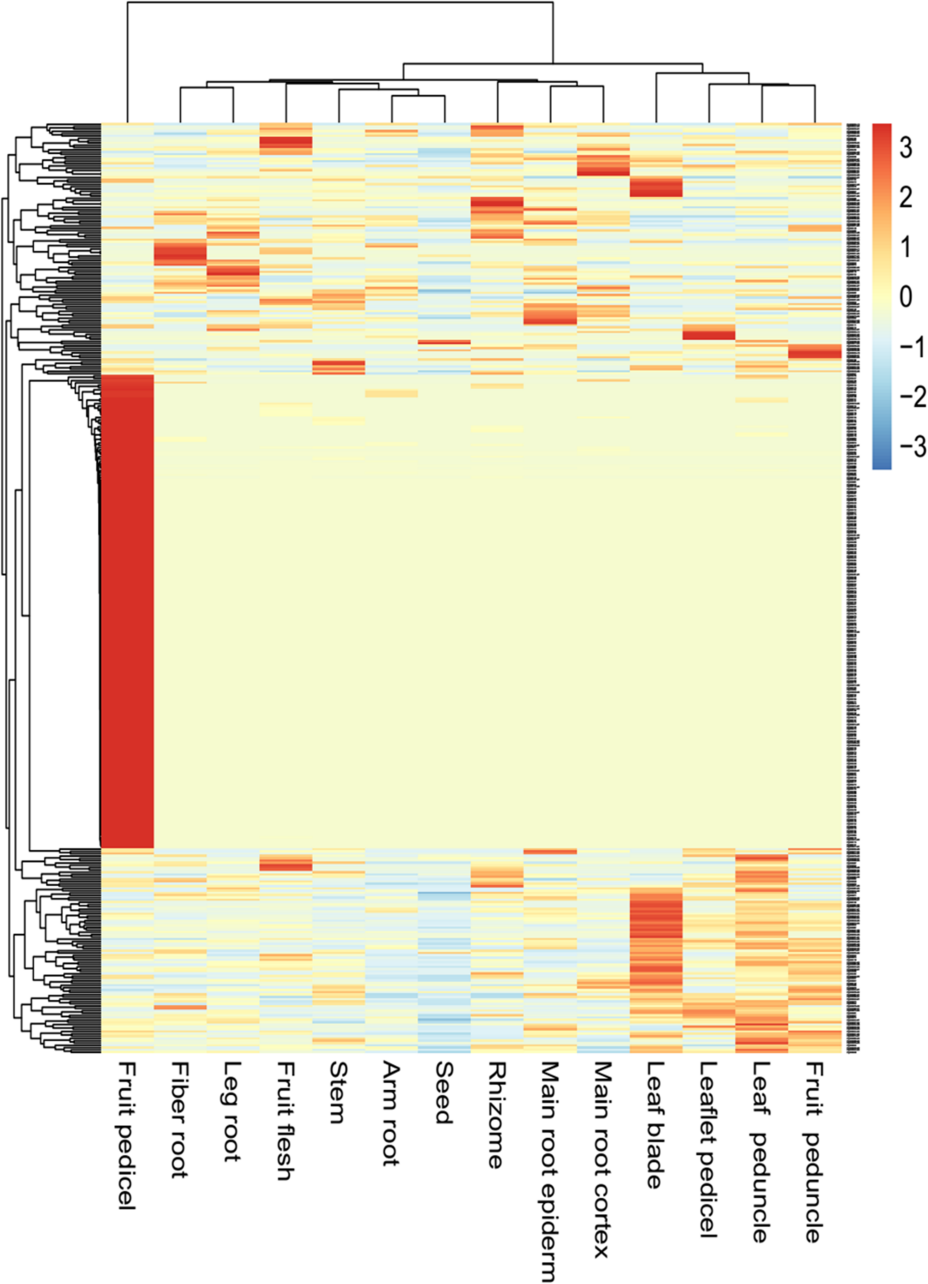
Expression heatmap of the *PgNBS* gene transcripts in different tissues of four year-old plants. Four hundred and three of the 412 NBS gene transcripts were found to be expressed in different tissues of the four-year-old plants and therefore, used for the heatmap construction.

**Fig 7:**
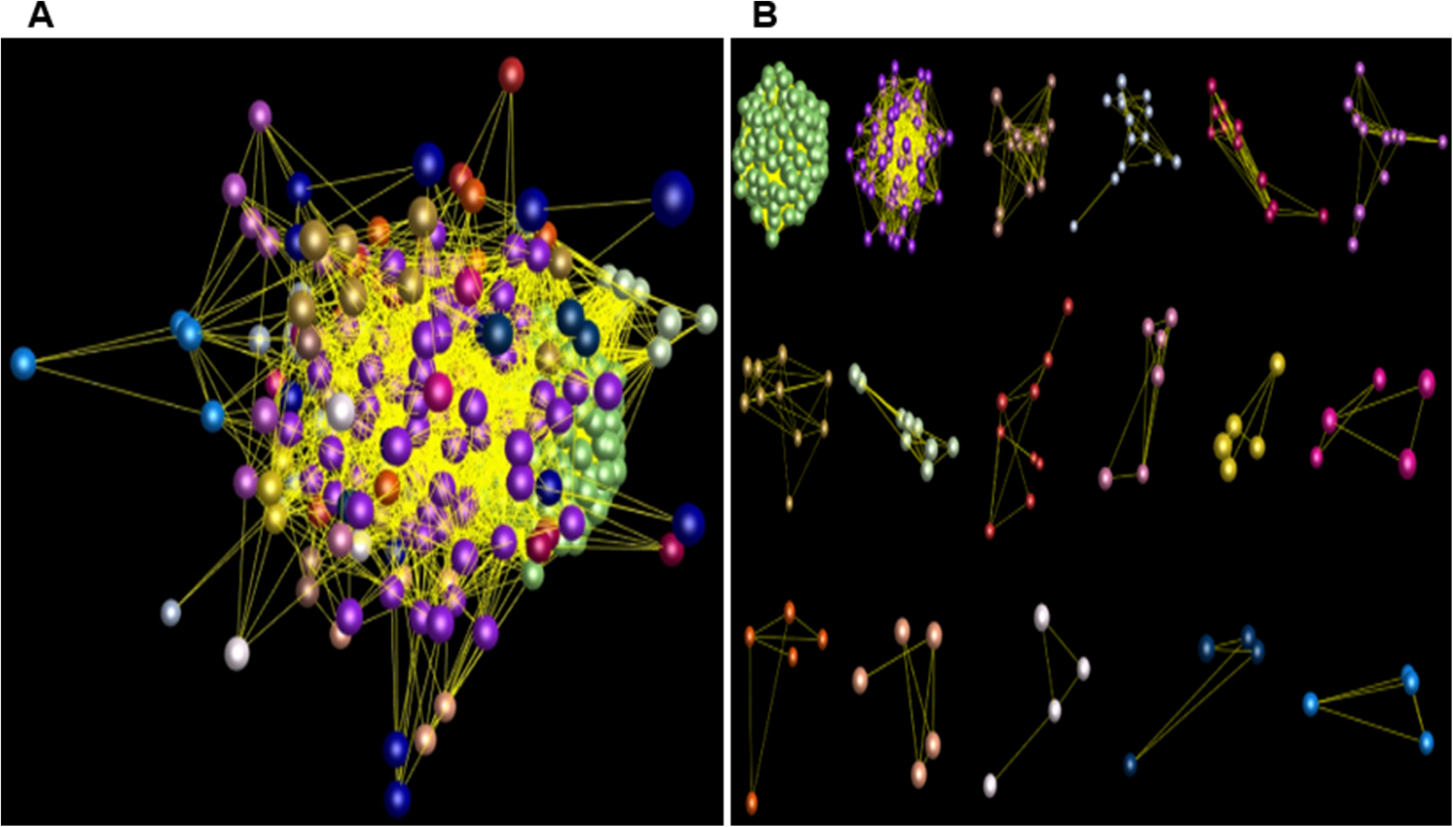
Co-expression network of the 403 *PgNBS* gene transcripts in ginseng different tissues. The network was constructed at a cutoff of *P* ≤ 0.05. (A) The overall view of the network consisting of all 403 transcript nodes indicated by colorful balls, 21,140 interaction edges indicated by the lines between the balls and 17 clusters indicated by different colors. (B) 17 different clusters constituting the network.

Comparative analysis showed that the numbers of the *PgNBS* gene transcripts categorized into each GO functional category (Fig 1B) varied, with a CV of from 25% to 374% (S2 Fig). The most consistent functional categories in number of transcripts among the 14 tissues were Small Molecule Metabolic Process (BP) and Intracellular (CC) (CV = 25%) and the most variable functional categories were Translation Factor Activity (MF), Nucleic Acid Binding (MF), GTPase Activity (MF), DNA Binding (MF), Ribosome (CC), Organelle (CC), Mitochondrion (CC), Cytosol (CC) and Translation (BP) (CV = 374%). The numbers of the *PgNBS* gene transcripts categorized into Response to Stress (BP), Ion Binding (MF) and Cell Death (BP) had a moderate variation with CV = 69% - 110%. Heatmapping showed that most (205) of the *PgNBS* gene transcripts (341) expressed in fruit pedicle were up-regulated, while only two of the transcripts expressed in arm root were up-regulated. From four to thirty-nine of the *PgNBS* gene transcripts were up-regulated in each of the remaining 12 tissues (Fig 6). The co-expression network analysis of the 403 *PgNBS* gene transcripts expressed in the 14 tissues resulted in a single network (*P* ≤ 0.05) that consisted of 403 gene nodes, 21,140 co-expressing edges and 17 clusters (Fig 7). The result showed that each of the *PgNBS* genes co-expressed with 2 to 199 other *PgNBS* genes, with an average of 105 other *PgNBS* genes, suggesting their co-originated functions.

### Expression characteristics of *PgNBS* gene transcripts in different aged roots

Next, we profiled the expressions, determined the GO functional categories and constructed the expression heatmap and co-expression network of the gene transcripts in the roots of 5-, 12-, 18- and 25-year-old ginsengs. One hundred fifty-two of the 412 *PgNBS* gene transcripts were found to express in the different year-old roots (Fig 5B and S6 Table). Forty-nine (12% of the 412 *PgNBS* gene transcripts) expressed in the roots of all four aged ginsengs, while also 49 (12%) expressed in the roots of only one aged ginseng. Thirty-three (8%) and 21 (5%) of the 412 *PgNBS* gene transcripts expressed in the roots of two or three of the four aged ginseng roots. Among the four aged roots, 95, 87, 93 and 98 of the *PgNBS* gene transcripts expressed in 5-, 12-, 18- and 25-year-old roots, respectively, suggesting that the numbers of *PgNBS* gene transcripts expressed were relatively consistent in different aged roots (CV = 5.0%).

**Fig 8:**
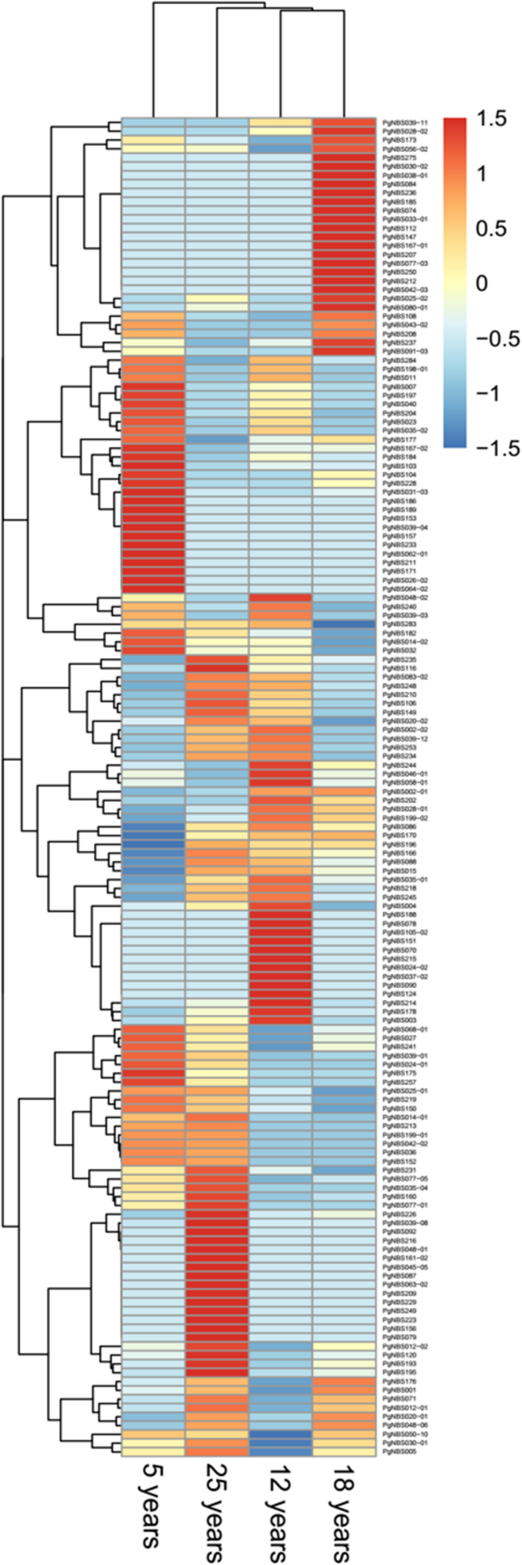
Expression heatmap of the *PgNBS* gene transcripts in the roots of different year-old plants. One hundred fifty-two of the 412 NBS gene transcripts were found to express in the roots of these different year-old plants and therefore, used for the heatmap construction.

GO comparative analysis showed that Anatomical Structure Development (BP), Cell Differentiation (BP), Embryo Development (BP), Reproduction (BP), Extracellular Region (CC), Intracellular (CC), Nucleus (CC) and RNA Binding (MF) had no difference in the number of the *PgNBS* gene transcripts into each functional category among the four aged roots (S3 Fig), while Biosynthetic Process (BP), Cellular Protein Modification Process (BP), Small Molecule Metabolic Process (BP) and Plasma Membrane (CC) had the largest variation in the number of the *PgNBS* gene transcripts into each functional category among the four aged roots, with a CV = 100%. Heatmap analysis revealed that when an expression level threshold of 1.5 was applied, 55, 30, 18 and 65 of the *PgNBS* gene transcripts were up-regulated in 5-, 12-, 18- and 25-year-old roots, respectively and the up-regulated *PgNBS* gene transcripts were largely different (Fig 8). The network analysis grouped 150 of the 152 *PgNBS* gene transcripts expressed in the four aged roots into multiple co-expression networks with a total of 542 co-expressing edges.

### Expression characteristics of *PgNBS* gene transcripts in four-year-old roots of different genotypes

**Fig 9:**
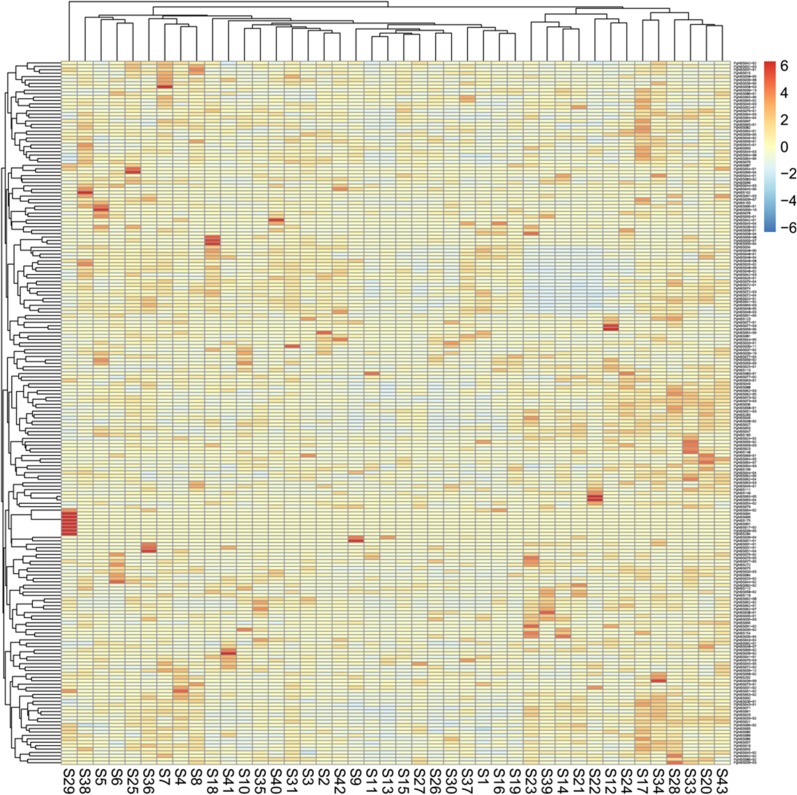
Expression heatmap of the *PgNBS* gene transcripts in the four-year-old roots of different farmers’ cultivars (e.g., S43). Two hundred and six of the 412 NBS gene transcripts were found to express in the roots of these cultivars and therefore, used for the heatmap construction.

**Fig 10:**
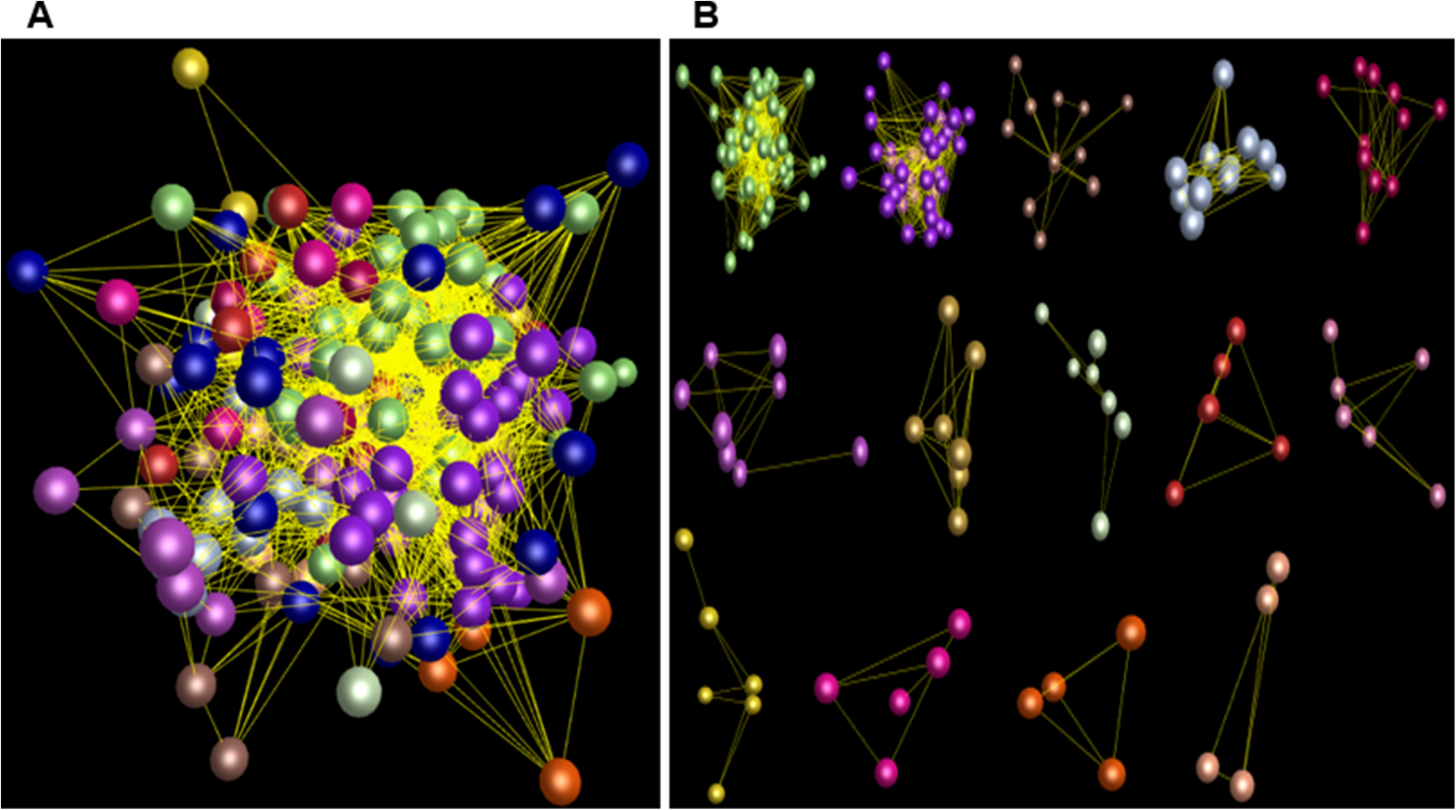
Co-expression network of the *PgNBS* gene transcripts in the 4-year-old roots of different ginseng genotypes. The network was constructed at a cutoff of *P* ≤ 0.05. (A) The overall view of the network consisting of all 206 nodes indicated by colorful balls, 2,446 edges indicated by the lines between the balls and 14 clusters indicated by different colors. (B) 14 different clusters constituting the network.

Finally, we profiled the expressions, determined the GO functional categories and constructed the expression heatmap and co-expression network of the gene transcripts in the 4-year-old roots of 42 farmers’ cultivars of Jilin ginseng. A total of 206 of the 412 *PgNBS* gene transcripts were found to express in the 4-year-old roots of these 42 farmers’ cultivars (S7 Table). Of the 206 *PgNBS* gene transcripts expressed in the 4-year-old roots of different genotypes, the numbers of the transcripts expressed in each genotype were relatively consistent, varying only from 102 to 140, with a CV = 6.0% and an average of 125. GO analysis showed that the numbers of *PgNBS* gene transcripts categorized into each functional category varied from CV = 0.0% to CV = 364.9%. Small Molecule Metabolic Process (BP), Cellular Component (CC) and Intracellular (CC) were consistent in number of *PgNBS* gene transcripts among the 42 genotypes (CV = 0.0%), while Carbohydrate Metabolic Process (BP), Cell Wall Organization or Biogenesis (BP), Signal Transduction (BP) and Kinase Activity (MF) showed the largest variation in number of *PgNBS* gene transcripts among the 42 genotypes (CV = 364.9%) (S4 Fig). Heatmap analysis did not formed distinct expression patterns characterizing each genotype, expect for genotype S29 that has a cluster of eight transcripts that were significantly up-regulated (Fig 9). Network analysis led to a single co-expression network of all 206 *PgNBS* gene transcripts expressed in the roots of the genotypes (Fig 10A). The network consisted of 206 gene nodes, 2,446 co-expressing edges and 14 clusters (Fig 10B). Each of the transcripts in the network co-expressed with 1–64 other *PgNBS* gene transcripts and an average of 24 other *PgNBS* gene transcripts.

## Discussion

### The *PgNBS* gene family is a size moderate, diverged and ancient NBS-encoding gene family

We have genome-wide identified and characterized the NBS-encoding genes expressed in Jilin ginseng. We identified 412 *PgNBS* gene transcripts from the transcriptomes of Jilin ginseng developed from 14 tissues [19]. These transcripts were derived from 284 gene models (Table 1), with an average of 93 gene models expressed in each tissue (S5 Table). According to a study in maize (X. Qi, M.P. Z., X. Su, J. Qin and H.-B. Zhang, unpublished) that approximately 30.7% of the NBS genes were expressed in a tissue, the number of *PgNBS* genes in the ginseng genome is estimated to be approximately 303. This number is much higher than that of *A*. *thaliana* (177) [6], but it is much lower than those of poplar (402) [16], rice (400–785) [12, 14, 30], cotton (1,350) and soybean (1,044) [30] that have smaller genomes than ginseng.

The *PgNBS* gene family has the typical features of the NBS gene family in dicot species, including both CC- and TIR-typed genes (Table 1). Although its genes maintain six or seven of the eight conserved motifs identified in the NBS domain of the NBS gene proteins in plants [11] and three or four conserved motifs in the N terminal region of the NBS gene proteins (Figs 2 and 3), they could be classified into TNL, CNL., TN, CN, N, NL, RPW8-NL and RPW8-N types (Table 1). However, the ratio of CNL-typed to TNL-typed differed from those of other dicot species. For instance, the ratio of the CNL-typed to TNL-typed for *A*. *thaliana* was approximately 1: 2 [6] and that for potato was about 2: 1 [11]. This study revealed that the ratio for Jilin ginseng is nearly 1: 1. Moreover, a majority (84.15%) of the *PgNBS* genes are characterized with the NBS-N domain. In addition, RPW8- typed genes were found in the *PgNBS* gene family, in which some new conserved motifs such as Motifs 5, 6 and 8 were found.

The *PgNBS* gene family originated at least before the separation between dicot and monocot plants (Fig 4). Of the 67 *PgNBS* genes phylogenetically analyzed, approximately 9% originated before dicot plants split from monocot plants and 34% originated before the Asterids split from the Rosids. Only about 15% of the *PgNBS* genes originated after *P*. *ginseng* originated.

### The *PgNBS* gene family has functionally dramatically differentiated, but expresses coordinately

Although the *PgNBS* genes identified in this study belong to a single gene family, they are functionally categorized into 36 functional subcategories of CC, MF and BP. It is recognized that they are mainly involved in four of the 36 functions, including Molecular_Function, Ion Binding, Cell and Response to Stress (Fig 1). In particular, the genes categorized into Response to Stress and Cell Death likely play a role in plant defense to pathogens. Nevertheless, this study shows that all the genes in the family co-express, regardless of in different tissues or in the roots of different genotypes, and form a single co-expression network (Figs 7 and 10). These results indicate that the genes of the *PgNBS* gene family, in spite of their functional divergence, still function in a coordinated manner.

### The genes of the *PgNBS* gene family express differently in different tissues, at different developmental stages and among different genotypes

This study shows that the expressions of most of the genes in the *PgNBS* gene family vary in multiple fold not only at the expression level, but also in the number of genes in different tissues (S5 Table), at different developmental stages (S6 Table) and among different farmers’ cultivars (S7 Table). Moreover, such large expression variations are also common among different *PgNBS* genes within a tissue, at a different development stage or in a particular genotype. A distinguishing character of the gene expression variation is that nearly 39% of 412 *PgNBS* gene transcripts identified in this study only express in a tissue or are tissue-specific and 12% express at a developmental stage or are developmental stage-specific (Fig 5). Some of the *PgNBS* genes may be up-regulated in a tissue, at a developmental stage or in a genotype, but down-regulated in the other tissues, at the other developmental stages or in the other genotypes (Figs 6, 8 and 9). Finally, the numbers of the *PgNBS* gene transcripts categorized into each functional category also vary dramatically across tissues, developmental stages or genotypes (S2–S4 Figs). These expression variations of the genes, therefore, are likely associated with the variation of their functions.

## Conclusions

We have identified 284 *PgNBS* genes that are expressed in Jilin ginseng and found that the members of the *PgNBS* gene family, in spite of their same origin, have dramatically differentiated in function and vary in spatial and temporal expression and network. These studies have revealed several new characters of the NBS gene family in plants in general and in Jilin ginseng particularly. These results have provided a deep insight into the origin, evolution, expression and function of the NBS gene family, and also resources and tools for further characterization of the genes that defense to pathogens in ginseng and for enhanced ginseng disease resistance breeding.

## Supporting information

S1 Table The expressed *PgNBS* genes of ginseng and their sequences. (XLSX)

S2 Table Annotation and GO functional categorization of the *PgNBS* gene transcripts. (XLSX)

S3 Table Predicted proteins of the 67 *PgNBS* genes with complete NBS domains used for gene phylogenetic analysis. (XLSX)

S4 Table The cloned R genes used as evolutionary controls for *PgNBS* gene family phylogenetic analysis. (XLSX)

S5 Table Expression profiles of the *PgNBS* genes in different tissues of four-year-old plants. (XLSX)

S6 Table Expression profiles of *PgNBS* genes in different year-old roots. (XLSX)

S7 Table Expression profiles of *PgNBS* genes in four-year-old roots of different farmers’ cultivars of Jilin ginseng (indicated by S1 through S43). (XLSX)

S1 Fig Fourteen tissues of four-year-old ginseng plants. (TIF)

S2 Fig Comparison of the GO functional categorization of the *PgNBS* gene transcripts among 14 tissues of four-year-old plants. (TIF)

S3 Fig Comparison of the GO functional categorization of the *PgNBS* gene transcripts among different year-old roots. (TIF)

S4 Fig Comparison of the GO functional categorization of the *PgNBS* gene transcripts among the four-year-old roots of 42 ginseng farmers’ cultivars. (TIF)
