# Supplementary figures and images for: Functional differentiation and spatial-temporal co-expression networks of the NBS-encoding gene family in Jilin ginseng, Panax ginseng C.A. Meyer
Source: PLoS One. 2017 Jul 20;12(7):e0181596. doi: 10.1371/journal.pone.0181596 (PMC5519184; doi:10.1371/journal.pone.0181596)

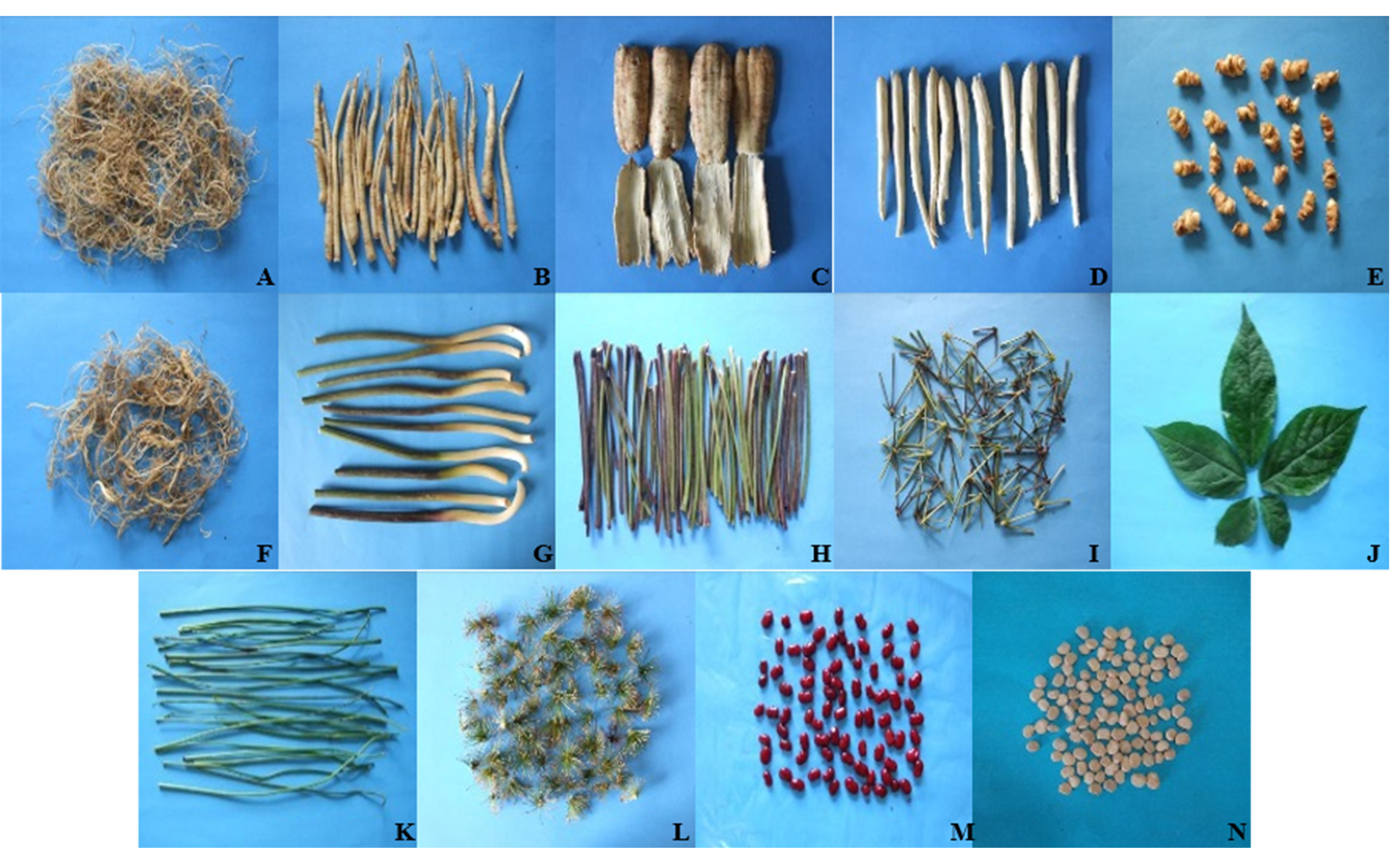

Supplement: S1 Fig — (TIF) [file pone.0181596.s008.tif]

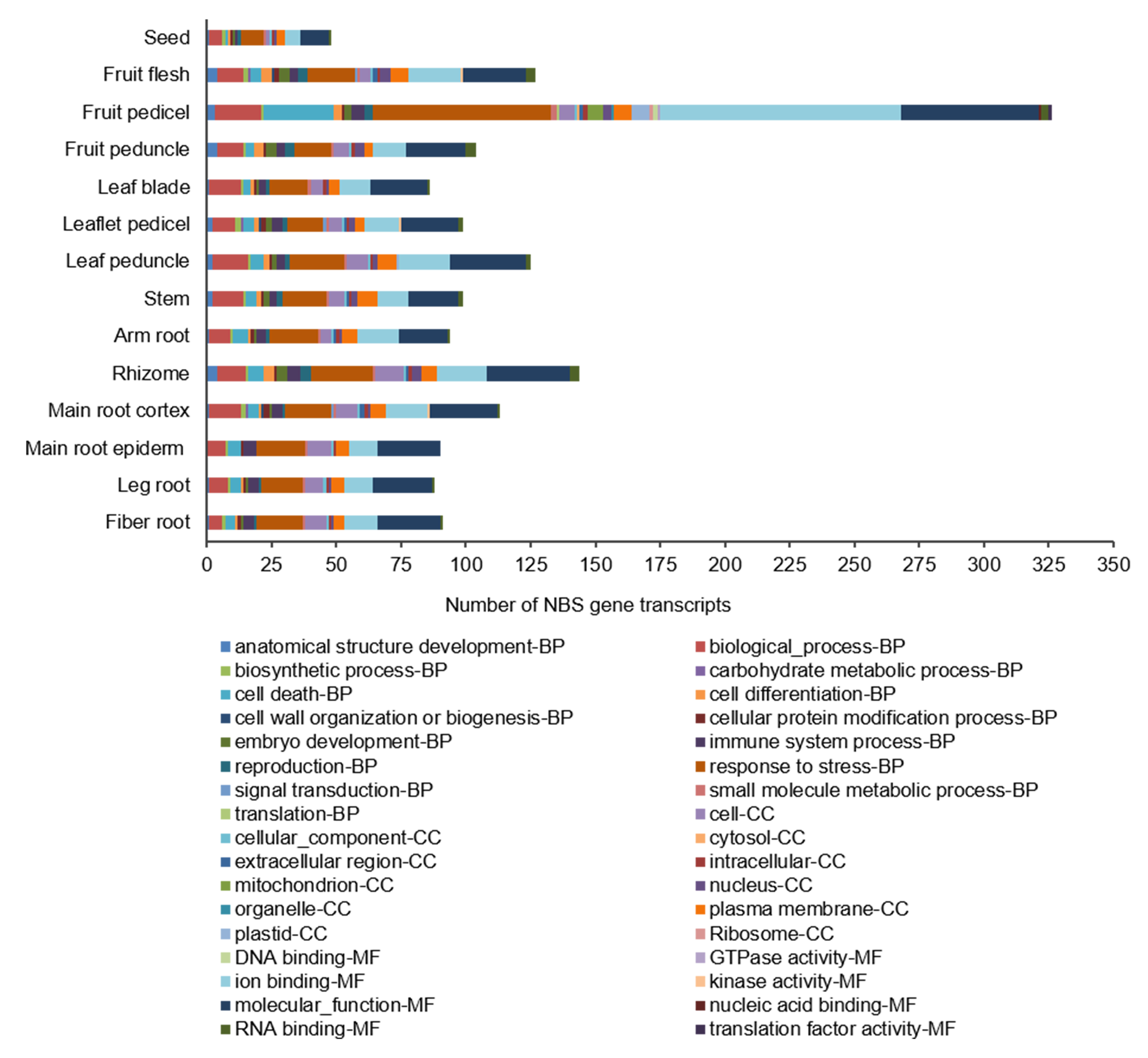

Supplement: S2 Fig — (TIF) [file pone.0181596.s009.tif]

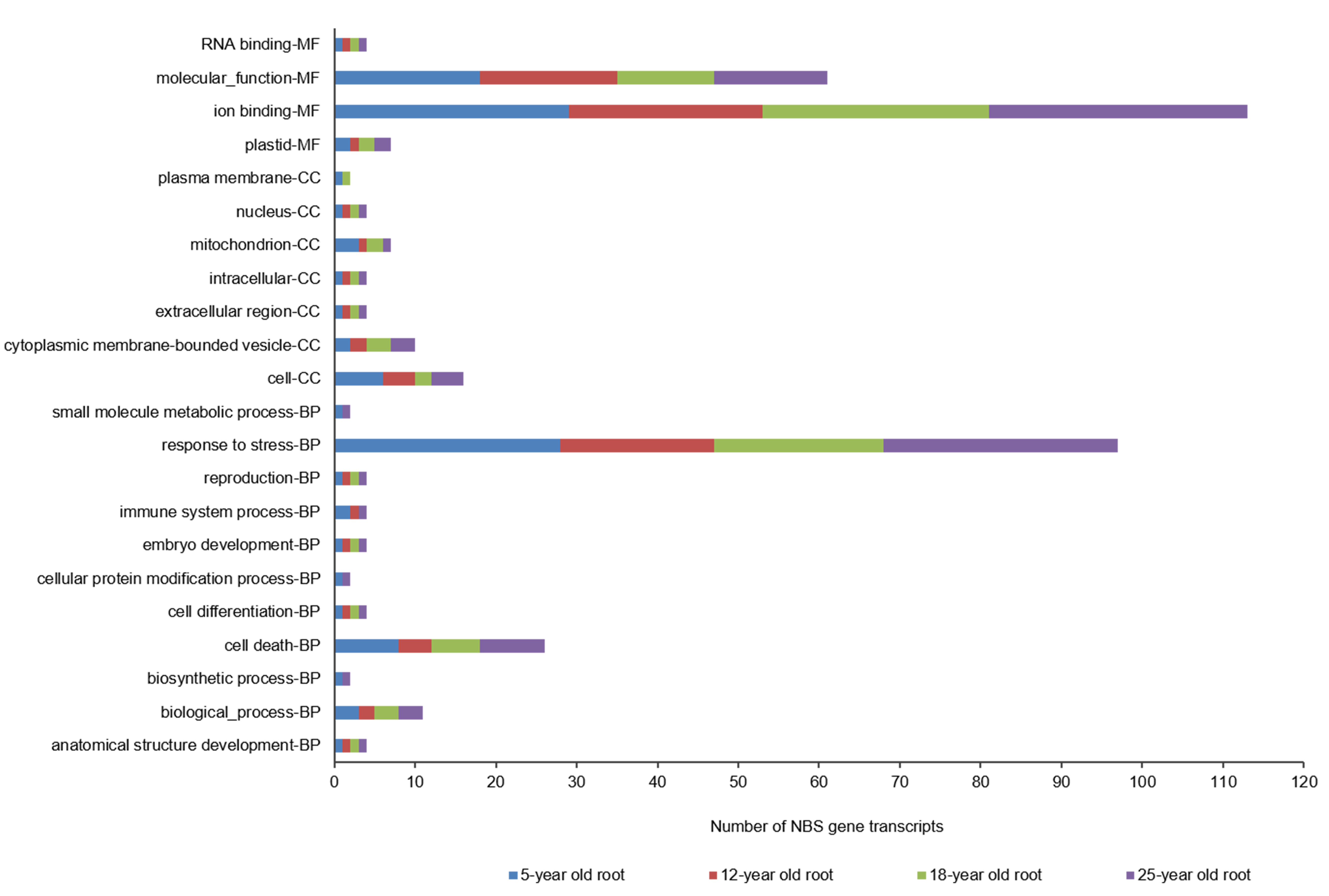

Supplement: S3 Fig — (TIF) [file pone.0181596.s010.tif]

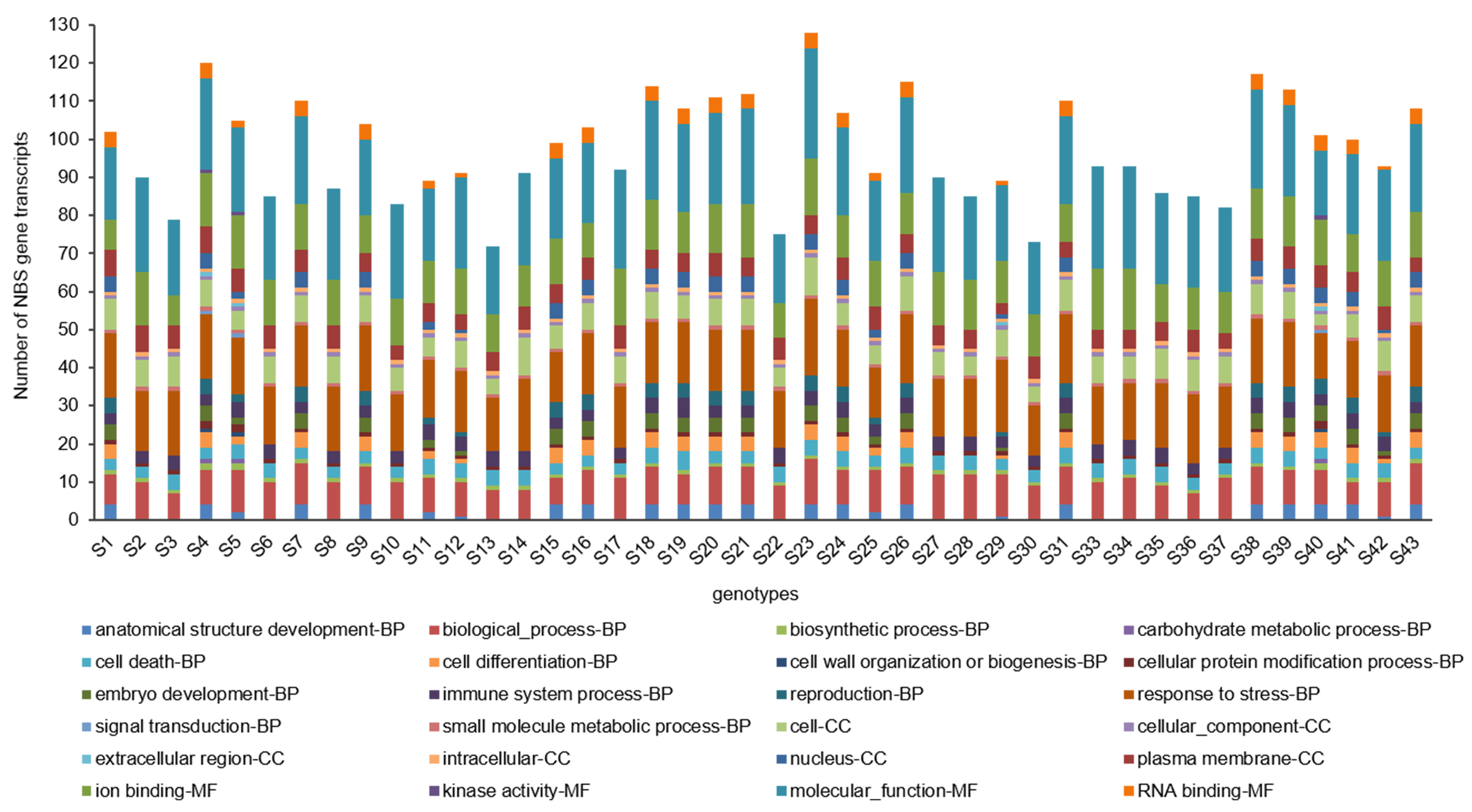

Supplement: S4 Fig — (TIF) [file pone.0181596.s011.tif]
